# Fouling and Chemical Cleaning of Microfiltration Membranes: A Mini-Review

**DOI:** 10.3390/polym13060846

**Published:** 2021-03-10

**Authors:** Aysegul Gul, Jakub Hruza, Fatma Yalcinkaya

**Affiliations:** Centre for Nanomaterials, Advanced Technology and Innovation, Technical University of Liberec, Studentska 1402/2, 46117 Liberec, Czech Republic; aysegul.gul@tul.cz (A.G.); jakub.hruza@tul.cz (J.H.)

**Keywords:** microfiltration, cleaning, chemical, membrane, antifouling, fouling

## Abstract

Membrane fouling is one of the main drawbacks encountered during the practical application of membrane separation processes. Cleaning of a membrane is important to reduce fouling and improve membrane performance. Accordingly, an effective cleaning method is currently of crucial importance for membrane separation processes in water treatment. To clean the fouling and improve the overall efficiency of membranes, deep research on the cleaning procedures is needed. So far, physical, chemical, or combination techniques have been used for membrane cleaning. In the current work, we critically reviewed the fouling mechanisms affecting factors of fouling such as the size of particle or solute; membrane microstructure; the interactions between membrane, solute, and solvent; and porosity of the membrane and also examined cleaning methods of microfiltration (MF) membranes such as physical cleaning and chemical cleaning. Herein, we mainly focused on the chemical cleaning process. Factors affecting the chemical cleaning performance, including cleaning time, the concentration of chemical cleaning, and temperature of the cleaning process, were discussed in detail. This review is carried out to enable a better understanding of the membrane cleaning process for an effective membrane separation process.

## 1. Introduction

In recent years, rapid industrialization and the increasing population have placed serious pressures on the balance of nature. The largest effects are manifested as a decrease in clean water resources, an increase in wastewater, and poorer water quality. The decrease in clean water resources has forced people to develop new ideal technologies.

Some of the current technologies for water treatments are oxidation, photocatalysis, Fenton/photo-Fenton processes, adsorption, biological treatment, electrochemistry, membrane filtration, and coagulation/precipitation. These applications have several disadvantages such as high cost [1,2].

Advanced oxidation processes (AOPs) are aqueous phase oxidation methods for the removal of the target pollutant [3]. Advanced oxidation processes are based on the production of hydroxyl (OH) radicals depending on the needs of the specific treatment [4]. Advanced oxidation processes are useful as they offer various methods for the production of OH radicals [5]. Generally, the installation of advanced oxidation processes is not cost-intensive [3]. Oxidation technologies use agents such as chlorine, ozone, and hydrogen peroxide. These chemicals generally have high costs. The disadvantage of using chlorine for oxidation is that as a gas it is highly toxic, corrosive, and produces toxic byproducts [6]. Furthermore, the total cost of this treatment is usually high [2,7].

Photocatalysis is based on the use of semiconductor particles, which have been demonstrated to efficaciously degrade pollutants. Titanium dioxide (TiO2) is the most commonly used material for photocatalysis. TiO2 is usually preferred due to being cheap, highly photoreactive, chemically and biologically inert, nontoxic, and photostable [8,9]. Photocatalysis is often limited by the turbidity of the water being treated, as the ultraviolet (UV) light must be able to penetrate it [1]. Another significant disadvantage is the limited life-span of the UV-lamp used [10].

Biological processes remove pollutants such as biodegradable organics, nitrate, synthetic organic compounds, iron, ammonia, and manganese. Biological applications have several limitations such as performance variability, start-up and maintenance, public health considerations, and release of microorganisms. The carbon source-to-nitrate ratio is an important criterion for application performance and should be carefully monitored. Microbial activity may be adversely affected by toxicants such as heavy metals. Start-up times are generally longer than those of other applications. Biodegradation products of biological applications (which are mostly unidentified) and their potential health effects are not yet known [11]. The most important issues with biological applications are that they are nonrapid processes (kinetic issues) with poor color removal and are accompanied by the weak biodegradability of certain molecules (dyes) (BAS) [12].

Adsorption is a surface phenomenon for the removal of organic and inorganic pollutants. The process is based on adsorbates and adsorbents, whereby adsorbates are defined as solutes retained on a solid surface and adsorbents are the surfaces on which the solutes are held. Adsorption is the surface storage of the adsorbate on the adsorbent [2]. Clogging of pores is the most significant obstacle faced by adsorption treatments. In addition, this method only removes the pollutant but does not transform it, which generates a hazardous waste stream [1,12].

Electrochemistry is based on electrochemical cells that do not use chemical reagents. Instead of reagents, the cells construct oxidizing species by the reactions that appear on the anode surface [13]. The high initial purchase cost of the equipment is a hindrance to the use of electrochemistry [12].

Coagulation/flocculation is a process used in water and wastewater treatment. The process is based on the addition of ferric chloride and/or polymers to the wastewater. The colloidal materials are then destabilized and cause small particles to accumulate in larger settable flocks [14,15]. The biggest obstacle is the difficulties in management, treatment, and cost resulting due to increased sludge volume [12].

Membrane separation technologies are quite remarkable. They have several advantages, such as decreased sludge generation, the superiority of permeate, and the possibility to completely recycle the water. Compared to conventional technologies, membrane technologies require less space, they are easier to use, and their operational costs are more manageable [16]. However, membrane filtration treatments have high investment costs for small and medium-sized industries. The greatest challenge of membrane technologies is to overcome the fouling issue, which reduces membrane performance and life-span.

Membranes provide a barrier for separating two phases and selectively limit the passage of various components [17]. Table 1 describes the membrane process.

Membrane separation technologies are divided into the following four processes based on pore size: microfiltration (0.1–5 µm), ultrafiltration (0.01–0.1 µm), nanofiltration (0.001 to 0.01 µm), and reverse osmosis (0.0001 to 0.001 µm) [18]. These processes are shown tabular in Table 2. They may also be classified as being either polymeric or ceramic-based on the materials used in their production [19].

Microfiltration (MF) is one of the most popular techniques for wastewater treatment. The history of microfiltration began in the 1920s and 1930s. The first commercially available membranes were collodion (nitrocellulose) membranes produced in 1926. The number of membrane producers increased during the 1940s; however, the use of microfiltration membranes was limited to laboratories and very small-scale industries until the mid-1960s. The most widely used process for microfiltration is dead-end or in-line filtration. During the 1970s, the use of membranes became possible in large-scale industries and an alternative process known as cross-flow filtration was introduced. In the mid-1980s, ceramic tubular cross-flow filters were made commercially available. In the next few years, semi-dead-end filtration emerged as a third type of microfiltration. The first-ever microfiltration/ultrafiltration systems were installed for surface water applications in 1990–1993 [20,21,22].

Microfiltration is commonly applied in pharmaceutical, food and beverage, and semiconductor industries [18]. Microfiltration uses the physical separation principle to remove micrometer-sized substances such as suspended particles, large bacteria, major pathogens, proteins, and yeast cells [21,22].

Microfiltration membranes offer the possibility to use a wide range of polymers such as polyvinylidene fluoride (PVDF), polysulfone (PS), polyamides (PA), cellulose acetates (CA), polytetrafluoroethylene (PTFE), olefins, or polycarbonate (PC). These polymers are good membrane materials due to their properties such as excellent film-forming properties, good mechanical strength, thermal and physical and chemical stability, as well as stability over a wide range of pH levels.

Membranes have several advantages such as no need to change the temperature and pH of the solution in separation by microfiltration. Microfiltration may also achieve without the addition of chemicals, whereby decreasing production costs, improving product quality and reducing labor costs [23].

Regrettably, membrane fouling or blockage remains a significant problem for the use of microfiltration. In this case, permeation flux is weakened, productivity is reduced, and the service life of filters is limited. This situation is an inevitable obstacle to the expansion of its application.

Fouling is the accumulation of particles, macromolecules, biomolecules, salts, and colloids, etc., on the membrane surface or within the pore structure. Recently many studies have focused on mainly oil [24,25], organic algae [26], proteins [27], colloidal material [28], and organic matter foulants [29]. Characterization of the foulants is important to determine whether they accumulate on the surface of the membrane or within the pores. Fouling is a complex phenomenon influenced by factors such as trans-membrane pressure [30], cross-flow velocity [31], and temperature [32], as well as feed characteristics [33] such as foulant form and size, foulant concentration, and feed pH, and membrane properties such as hydrophilicity/hydrophobicity, roughness, pore size, and pore type.

The performance of membranes in microfiltration is highly related to their fouling and researchers have improved various strategies for its reduction. One of these strategies is cleaning in a membrane unit. Membrane cleaning aims to maintain or restore membrane performance, for example, permeability or selectivity, which may alter due to membrane fouling. It would be good to note that most of the studies on membrane cleaning in recent years are related to ultrafiltration and that the work done on microfiltration is rather limited. The current results on the membrane cleaning process are shown in Table 3.

Chemical cleaning may be regarded as an integral part of membrane operation and must be performed regularly to remove fouling to ensure product safety and continuous operation. The literature on chemical cleaning in microfiltration has a rather narrow scope and no comprehensive study on chemical cleaning in microfiltration has been found. In this review, we try to summarize the membrane fouling and most common chemical cleaning methods of microfilters. First of all, the common foulants and fouling mechanisms are described, then the suitable cleaning methods are reviewed. The chemicals used during the cleaning have huge effects on the membrane structure, surface, and deterioration. For this reason, suitable chemicals for the most common membranes are also mentioned. Lastly, effective parameters, such as temperature, cleaning time, pH, etc. are reviewed.

## 2. Membrane Foulants and Affecting Factors of Fouling

### 2.1. Foulants and Mechanism of Fouling in Microfiltration Membrane

**Figure 1:**
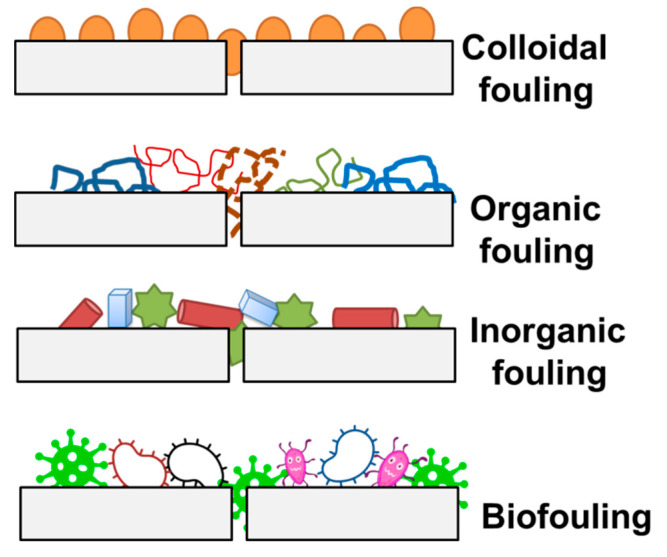
Schematic illustration of colloidal, organic, inorganic, and biofouling.

Several types of membrane fouling have been identified based on the chemical nature of foulants, membrane process, and types of foulants as well as their interaction with the membrane surface. Microfiltration fouling may include (a) inorganic fouling/scaling, (b) particle/colloidal fouling, (c) microbial/biological fouling, and (d) organic fouling [23,24]. In detail: (a) inorganic fouling is the accumulation of inorganic precipitates such as metal hydroxides on the membrane surface or within pore structures, (b) particulate/colloidal fouling includes algae, bacteria, some natural organic matter, and colloids, (c) biofouling, which is the formation of biofilms on the membrane surface. As a result of microbial activity, these biofilms (algal, bacterial, or fungal) form and extricate biopolymers (polysaccharides, proteins, and amino sugars), (d) organic fouling includes proteins, amino sugars, polysaccharides, and polyoxy aromatics [24,25]. Figure 1 illustrates these four fouling types, and Table 4 shows the fouling mechanism of fouling types [41].

The common foulants encountered in microfiltration applications are summarized in Table 5.

The foulants interact with the membrane surface both physically and chemically, but chemical interaction between membrane surface and foulants can degrade the membrane material. Hydrophobic interactions, hydrogen bonding, van der Waals attractions, and extracellular macromolecular interactions cause irreversible flux decrease in water treatment applications. The interactions between feed materials and membranes are often affected by the polymer’s molecular size and charge. A reversible flux decline is induced by concentration polarization, which accumulates solutes and particles on the membrane surface in a condensed boundary layer or liquid film, while an irreversible flux decrease is caused by fouling [20,35]. Unlike the irreversible flux decline, reversible flux decline can be eliminated by the physical cleaning process.

In colloidal fouling, the particles can physically affect the membrane surface and block the pores. It can also cause the formation of a cake layer and prevent transport to the surface. Organic foulants would be attached to the membrane by adsorption. Inorganic foulants are more prone to precipitation on the membrane surface due to pH change or oxidation. Bio foulants cause the formation of biofilm by sticking to the surface of the membrane. Proteins, as hydrophobic compounds, are more readily adsorbed on hydrophobic membranes’ surface than hydrophilic solutes due to hydrophobic interactions.

In contrast to a hydrophilic membrane, removing the adsorbed layer from a hydrophobic membrane is more complicated. Biofouling of membranes is also a serious issue; the slightly negative charge of bacteria combined with the hydrophobicity of cells results in the creation of a biofilm gel layer. Biofilm formation on the membrane surface is the result of adhesion and may cause membrane fouling. Biofouling decreases membrane efficiency by lowering precise membrane flux [20]. The physical cleaning process is not enough to remove biofouling. In this case, the chemical cleaning process is highly demanded to extend membrane efficiency and life-span.

**Figure 2:**
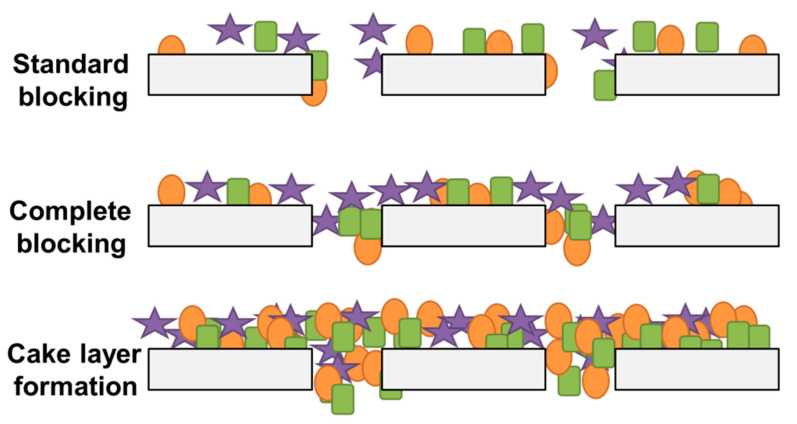
Membrane fouling mechanisms in microfiltration. Standard clocking d_foulant_ < d_pore_, complete blocking d_foulant_ > d_pore_, cake layer formation d_foulant_ > d_pore_ where d is membrane pore diameter.

To better understanding of fouling mechanisms, we can classify it as (1) standard blocking, (2) complete blocking, and (3) cake formation (solute aggregation) [47] which is shown in Figure 2 [48].

The size of the membrane pore is also significant in explaining the fouling mechanism. If the molecules fed by the membrane pores are similar, these molecules may cause partial clogging of the membrane pores. If the membrane pores are larger than the fed molecules’ size, these particles settle into the membrane pores and cause irreversible fouling. In cases where the membrane pores are smaller, the molecules gather on the membrane surface and cause the membrane pores to clog or form a gel layer.

Standard blocking is observed where the particles gather on the inner pore walls and lead to a reduction in pore size with time. Complete blocking occurs in which each particle, upon arriving at the membrane surface, experiences occlusion of pores by particles with no superposition of the particles because the particle size is larger than the pore area. Cake layer formation is observed where each particle locates on the other deposited particles. This means that there is no room for particles to directly obstruct the membrane area [35,36]. The general effects of cake layer accumulation, adsorption, and concentration polarization cause fouling. Combining physical and chemical cleaning methods, it is possible to reduce membrane fouling. First, we need to clarify the effective factors behind the membrane fouling.

### 2.2. Affecting Factors of Membrane Fouling

Affecting membrane fouling factors may be classified as membrane properties, solution (feed material) properties, and operational conditions. It is necessary to understand each factor clearly for an effective cleaning method. Herein, we tried to explain each factor under a different subtitle.

#### 2.2.1. Membrane Properties Effect on Membrane Fouling

Membrane morphology, such as pore size, pore size distribution, and pore geometry, has a major impact on fouling. Each morphology determines the predominant fouling mechanisms, such as the blocking of pores and the formation of cakes. One of the primary membrane properties is hydrophilicity/hydrophobicity. Hydrophilic membranes are thought to have a lower membrane fouling potential than hydrophobic membranes [49]. A membrane may be attractive or repulsive to water in an aqueous environment. The membrane composition and the corresponding surface chemistry determine the water interaction and thus influence its wettability. Membranes with groups capable of bonding hydrogen to water are hydrophilic and can absorb water. Hydrophobic membranes do not have active groups to form hydrogen bonds and have little or no tendency to absorb water. In this case, their tendency to contamination is increasing. Particles that contaminate membranes in aqueous environments tend to be hydrophobic. They tend to become clusters or group together to form colloidal particles because this process lowers the free interfacial energy. Greater membrane hydrophilicity is usually associated with higher charge density on the membrane surface. Fouling can thus be reduced by using membranes with their surface chemistry changed to make them hydrophilic. This hydrated surface will minimize fouling by preventing the absorption and deposition of hydrophobic foulants onto the membrane surface [50,51].

#### 2.2.2. Solution Properties Effect on Membrane Fouling

Some of the feed properties that affect fouling are solid (particle) concentration, particle properties, pH, and ionic strength. The increase in the concentration of feed causes a decline in the permeate flux. It is because the increase in the concentration of pollutants is responsible for the increase in membrane fouling. Particle size is also important to the understanding mechanism of fouling. Depending on the particle size, the particles can cause fouling by blocking pores, narrowing pores, or forming cakes. Some other factors are also important, such as pH, ionic strength, and particle electric charges. The charge on the membrane, the charge on the particles, conformation, and stability of, and thus adhesiveness of particles/molecules, as well as the size of the cake, are all influenced by the pH and ionic strength of the feed [38,42].

#### 2.2.3. Operational Conditions Effect on Membrane Fouling

The studies conducted to examine the effects of temperature, transmembrane pressure (TMP), and cross-flow velocity (CPV) on permeate flux are among the operating conditions. Membrane fouling can be minimized to some degree by improving operating conditions, but it is still unavoidable during the filtration phase.

Temperature impacts have been investigated, and results showed that the permeate flux increased with a low degree of fouling at higher temperatures [29]. Changing the feed temperature between 20 °C and 40 °C means that the penetrating flux rises to 60% [52]. Ren et al. [53] investigated a rising flux trend with increasing temperature, which was probably caused by the viscosity decline due to the temperature change and increased diffusion capacity of the feed water.

Some studies showed that improving CPV helped slow membrane fouling and increased flux. Higher cross-flow velocity mixing leads to a decrease in the combination of feed solids in the gel layer mainly because these components become more widely diffuse back towards a bulk, which leads to a reduction in the overall concentration polarization effect [41,54].

Controlling the transmembrane pressure (TMP), which is the difference in pressure between the feed and permeate streams, is important because it directly affects the permeation rate. The force of the fluid flowing into the membrane increases as the TMP rises, resulting in a higher permeate flux [42,55].

## 3. Microfiltration Membrane Cleaning Methods

Membranes lose their efficiency over time depending on their type, feeding materials, and process conditions. The most significant issue for membrane systems is fouling [56]. Cleaning is the most commonly used method for resolving this issue.

Membrane cleaning can be done in two ways based on fouling removal mechanisms: physical and chemical cleaning. In this section, physical cleaning and biological cleaning will be briefly mentioned, and chemical cleaning will be emphasized.

### 3.1. Physical Cleaning of Membranes

The removal of foulants on the membrane surface by applying hydraulic or mechanical forces is called physical cleaning. The most widely used physical cleaning methods are: hydraulic (forward and reverse flushing, backwashing, membrane relaxation, and air flushing) and mechanical (sponge ball and fluidized particle cleaning) methods. In addition to these methods, innovative methods such as ultrasonic and electric fields have been developed in recent years [57]. The basis of these methods is the mechanical processes applied to remove contaminants from the membrane surface. Each physical cleaning method is discussed in more detail in the following sections.

#### 3.1.1. Forward and Reverse Flushing

Reverse flushing is based on changing the permeate flush direction in the forward and backward direction for a certain time. This method is mostly used to remove colloidal particles from the membrane surface. Forward flushing is accomplished by pumping permeate water into the feed section at a pressure high enough for the pollutant to leave the membrane surface. Due to the faster flow and the resulting turbulence, the particles absorbed into the membrane are released and discharged [43,58].

#### 3.1.2. Air Flushing

Air Flushing can be applied simultaneously with filtration to reduce clogging or periodically to scrape the residues formed. The roles of air are loosening foulant from the fiber walls (suitable for hollow fiber configuration). With this method, the highest efficiency is achieved in a flat plate and tubular membranes, while the efficiency is less in hollow fiber and spiral-wound membranes [59].

#### 3.1.3. Backwashing

Backwashing is defined as the reverse filtration process in which the filtered material flows from the membrane to the concentrate side. The pressure on the membrane’s permeate side exceeds the pressure in the membranes, resulting in the purification of the pores [60]. Backwashing is easy to implement by adding a pump to the permeate side to reverse the flow, and chemicals can be used to increase backwashing cleaning performance (Chemically Enhanced Back Wash). Backwashing performance will be reduced if the membrane has a high internal pressure drop. Backwashing has a range of drawbacks, one of which is permeating for membrane cleaning, which is costly for production.

#### 3.1.4. Relaxation

The membrane relaxation method is based on diffusive back transport of foulants away from the membrane surface under a concentration gradient [61]. When air scouring is applied during relaxation, the removal efficiency of this method can also be increased.

#### 3.1.5. Sponge Ball

The sponge ball method is a system in which a sponge ball made of polyurethane or other materials is inserted into the permeator for a few seconds to remove the contaminant from the membrane surface. However, this system can only be applied to tubular modules [37,39].

**Figure 3:**
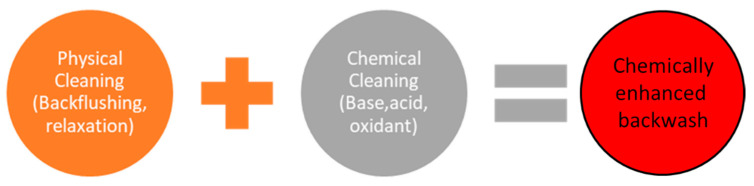
Membrane cleaning methods; physical cleaning, chemical cleaning, and their combination (chemically enhanced backwash).

These methods require complex equipment control and design during the implementation phase. In physicochemical methods, chemical agents are added to increase the physical cleaning efficiency [59]. Figure 3 illustrates cleaning methods.

Physical cleaning is a faster process than chemical cleaning and takes no more than two minutes. It does not require chemicals and does not produce chemical waste. It is less damage to the membrane. However, it is a less effective method compared to chemical cleaning [62]. While physical cleaning is only a solution for reversible contamination, nonreversible contamination requires chemical cleaning.

### 3.2. Chemical Cleaning

Chemical cleaning is defined as the removal of impurities by chemical agents, whereby restoring the membrane flux [30,42]. Permeability and selectivity, required for good flux performance and product quality control, are the most important determinants for the effectiveness of membranes. Chemical cleaning is also the most efficient method for restoring and maintaining these two important factors [28,42]. Due to this, chemical cleaning needs to be effective in order to separate the various types of foulants from the membrane, whereby restoring the permeate flux characteristics of the membrane.

#### 3.2.1. Chemical Cleaning Process

Microfiltration membranes are inclined to fouling; therefore, cleaning is required to control inorganic, microbial, biological, and colloidal fouling. Chemical cleaning is generally required when backwashing is unable to remove the foulants or restore the membrane flux.

Chemical cleaning treatments may be divided into the following basic types [63];

- Fouled membranes immersed in chemicals, such as “clean-in-place” (CIP),
- Soaking of fouled membranes using high-concentration cleaning agents using separate tanks, such as “clean-out-off-place” (COP),
- Adding chemicals to the feed stream, such as “chemical wash” (CW),
- Combining physical and chemical cleaning, such as “chemical enhanced backwash” (CEB).
- Chemical cleaning processes take place in six stages [41,47,56].
- Bulk reactions for cleaning reagents,
- Transport of chemical agent to the membrane interface,
- Transport of chemical agent into the foulant layer,
- Cleaning reactions in the fouling layer,
- Transport of cleaning reaction products back to the interface,
- Transport of product to the bulk solution.

These six stages may not always be required, and some stages may be skipped during the cleaning process [41]. The effectiveness of cleaning is measured based on how much membrane permeability can be restored and how fast the restoration of membrane permeability can.

To resolve the mechanisms of fouling and chemical cleaning, it is important to understand that interactions between foulant-foulant, foulant-membrane, and foulant-cleaning agent occur at the molecular level [64].

Liu et al. [47] stated that electrostatic interaction and hydrophobic/hydrophilic interaction between membranes and fouling materials have an impressive effect on membrane fouling. Outcomes of membrane fouling and chemical cleaning efficiency are directly related to that balance between hydrophobic adhesion and forces of electrostatic repulsion [47,55,65].

Membranes and fouling materials mostly carry a negative charge; therefore, electrostatic repulsion has an effect on decreasing fouling [25,58]. Based on this, the efficiency of chemical cleaning may be significantly increased by the use of electrostatic repulsion.

Hydrophobic interaction is based on the “like attracts like” principle. Membranes and solutes that have similar chemical structures have a tendency to attract each other [47]. When the hydrophobic adhesion overcomes the electrostatic repulsion, adhesion occurs on the membrane surface. Hence, the hydrophobic attraction may be the main cause of fouling [43,44]. Therefore, when selecting a chemical agent, its features should be carefully considered.

#### 3.2.2. Performance of Cleaning Agents in Microfiltration

Cleaning agents alters foulants on the membrane surface in the following three ways [63];

- The removal of foulants is possible,
- The morphology of foulants may change (swelling, compaction), and/or
- The surface chemistry of foulants may be changed by using hydrophobicity or an electrical charge.

Hydrodynamic conditions are required to promote contact between the chemicals and the foulants. Membrane cleaning involves the mass transfer of chemical agents to the fouling layer and reaction products back to the bulk liquid phase [50].

The selection of the optimal cleaning agent choice is based on the characteristics of the feeding materials. The morphology and structure of the foulants on membranes or within pores are important in determining the optimal chemical agent and cleaning conditions. The selected cleaning agent should be able to dissolve most of the foulants on the surface and remove them from the surface. Furthermore, the chemicals should not damage the membrane. Chemical cleanings agents may be classified into the following categories: caustics, alkalis, acids, enzymes, surfactants, sequestrants, and disinfectants, as shown in Table 6.

Obeidani et al. [68] studied the removal of oil from contaminated seawater using hollow fiber microfiltration. This study includes alkaline and acidic cleaning. Caustic soda, oxalic acid, and sodium hypochlorite have been used as chemical agents. The results showed that acid-based agents were more effective than their alkaline counterparts. Caustic soda was not found to be effective for flux recovery. Oxalic acid was found to be more effective than caustic soda. When the selected acidic cleaning agents, NaOH, and oxalic acid, were compared, their effectiveness was found to be the same.

Garmsiri et al. [34] investigated the influence of different chemical agents used to clean mullite ceramic microfiltration membranes used to remove oil contaminants from wastewater. The following cleaning steps were used: (i) forward water flushing; (ii) cleaning with chemical agents; and (iii) cleaning using vinegar and bicarbonate sodium. In the chemical cleaning process, an acid (H2SO4), surfactant (SDS), chelating agent (EDTA), and alkaline (NaOH) were used as cleaning agents as single, binary, and ternary compounds. SDS as the single compound was found to be the best cleaning agent with the highest flux recovery (57.78%). Of the binary compounds, SDS + EDTA was the best with the highest flux recovery. The ternary compounds SDS + NaOH + EDTA had the best flux recovery. The weakest chemical agent in this study was sulfuric acid.

Madaeni et al. [58] used HCI, NaOH, and Triton-X100 as chemical cleaning agents to remove whey protein from PVDF microfiltration membranes. All of the cleaning solution concentrations were 2%. The most effective agent was found to be Triton-X100, which is a surfactant. Triton-X100 showed a flux recovery of 4% and maximum resistance of removal of 86%. HCI had a moderate effect and NaOH had the weakest effect on cleaning.

Blanpain-Avet et al. [69] investigated the effect of multiple fouling and cleaning cycles on tubular ceramic microfiltration membranes fouled by whey protein concentrate. They applied a two-stage chemical cleaning process using solutions of NaOH (1 wt %) and HNO3 (5 wt % 0.08 M). Alkaline (NaOH) and acid (HN03) cleaning steps were applied with the permeate side open. Both the permeate and retentate were returned back to the cleaning solution tank. While NaOH was effective in flux recovery, citric acid was found to have a negative effect on membrane resistance.

Makardij et al. [70] used sodium hydroxide as a cleaning agent to remove skimmed milk residues from PVDF microfiltration and ultrafiltration membranes. Firstly, they used chemical pretreatment, which was conditioned by circulating a 0.05 M solution of sodium nitrate to improve flux. After conditioned, they used chemical cleaning. As another option, they cleaned the membranes by recirculating 0.5% hydrochloric acid for 30 min and subsequently washed them with deionized water for 10 min. Under the same cleaning conditions, they performed several different cleaning trials using a 0.5% citric acid solution. They observed that washing the membranes clogged with milk residue with deionized water was a very successful method of cleaning. The selected cleaning agents were found to damage the membranes.

Maskooki et al. [45] used ultrasonic waves together with two concentrations of EDTA as a chelating agent to reconstruct the flux of PVDF microfiltration membranes fouled by milk. In this study, they examined flux recovery, cleaned membrane resistance, and cleaning efficiency among their interaction effects. They tested mixed wave ultrasound at frequencies of 28, 45, 100 kHz. However, they used two different concentrations of EDTA, 1 mMole and 3 mMole. The results showed that mixed wave ultrasound has a higher cleaning efficiency than other treatments separately and in combination with 1 mMole EDTA. They also showed that ultrasound has a synergistic effect when used with EDTA as a cleaning agent.

Madaeni et al. [71], studied the cleaning optimization of PVDF microfiltration membranes fouled with raw milk residues. They focused on parameters including temperature, cleaning time, concentration, and cross-flow velocity of cleaning efficiency. In this study, they used nitric acid, hydrochloric acid, sulfuric acid, sodium hypochlorite, sodium hydroxide, potassium hydroxide, ammonium hydroxide, ammonia, EDTA, sodium dodecyl sulfate (SDS), triton X-100, phosphoric acid, and 2-propanol as chemical agents. They determined that EDTA was not effective at any concentration. Therefore, it was not recommended as a cleaning agent. It was reported that EDTA is suitable for combining with a base material. SDS was found to show effective results in combination with NaOH (as the cleaning agent) and EDTA (as the chelating agent). The study states that hydrochloride and nitric acids are not effective cleaning agents. On the other hand, bases are said to be able to saponify fat and dissolve proteins to a certain extent. It was found that sodium hypochlorite acted as a strong base.

Hou et al. [72] developed a kinetic model to calculate total membrane fouling resistance in chemical cleaning. They used NaOCI, SDS, and NaOH as the chemical agents to clean PAN microfiltration membranes fouled by activated sludge suspensions. They stated that NaOCl had the best cleaning performance and the highest Jr (83%), followed by SDS (62%) and finally NaOH (57%). Compared to the NaOH and SDS solutions, they stated that NaOCl was able to deactivate chlorella cells and bacteria and remove accumulated extracellular polymeric substances from the membrane surface.

Gan et al. [55] studied a synergetic cleaning procedure for ceramic microfiltration membranes fouled by beer. They used NaOH, HNO3, H2O2, and Ultrasil 11 as chemical cleaning agents. They postulated a three-step cleaning mechanism. After the beer filtration process, the membrane system was rinsed in place with water. To ensure that the membranes no longer contain beer and unstable surface deposits, the membranes were removed from the filtration system, mounted on another device, and further cleaned with distilled water. For chemical cleaning, the chemical agents were dissolved in distilled water and the solutions were preheated to a predetermined cleaning temperature. In combined simultaneous caustic cleaning and oxidation, the cleaning solution contained both NaOH and H2O2. They found that NaOH has the highest cleaning efficiency (FRstat ˆ0.64). HNO3 used in acidic cleaning was found to have a very weak effect (FRstat ˆ0.43). They suggested that there were no lipids amongst the known fouling constituents. This study emphasized that a successive two-stage cleaning process is more effective than single-stage caustic cleaning, but its operation is cumbersome. The cleaning solution was created at the optimum concentrations to contain both NaOH and H2O2. This method was demonstrated to be approximately 15% more effective by the CSCCO (combined simultaneous caustic cleaning and oxidation) process in FR stat Tcˆ808C than single-stage caustic cleaning.

Woo et al. [73] performed an investigation of the relationship between chemical agents and flux recovery for PVDF microfiltration membranes. In this study, foulants were selected as the organic matter (15 mg/L humic acid), inorganic matter (1 mg/L Fe and 1 mg/L Mn), and a combination of organic and inorganic matter (humic acid, Fe, and Mn). Backwashing, flushing, and chemical cleaning (1% sodium hydroxide solution and 2% citric acid solution) methods were used as the cleaning procedures. Changing the cleaning sequence of NaOH and HNO3 caused a change in the chemical cleaning efficiency (acid/base and base/acid). Flux recovery was 20% higher in the base/acid sequence. The results showed that the final fluxes were reduced by 8% for inorganic matter and by 78% for organic matter.

The cleaning methods of various membranes according to the type of foulants are given in Table 7.

## 4. Parameters Affecting Cleaning Efficiency

The efficiency of chemical cleaning is affected by the types of chemical agents used, as well as operational parameters such as time, temperature, and concentration. The operating conditions are necessary to achieve optimum cleaning efficiency.

### 4.1. Cleaning Time

The duration of chemical cleaning has not been adequately discussed in the literature. During the cleaning, sufficient time is required for the reaction of chemicals agents with the precipitated materials. Flux recovery increases by increasing the cleaning time at the early stage of cleaning. However, the efficiency is slowly reduced due to the limited capability of the chemicals to dissolve the deposited layer [71].

Madaeni et al. [71] studied the cleaning optimization of PVDF microfiltration membranes and determined that the cleaning time plays a crucial role in flux recovery. A longer cleaning time provides a higher flux recovery. However, they pointed out that these effects were limited. Madaeni et al. [58] achieved the same results in another study on whey protein cleansing.

V.Gitis et al. [87] proposed a definition for cleaning intensity, CT, that is the product of the cleaning time (t) and the concentration of the cleaning agent (C). They used polyethersulfone (PES) and PVDF membranes through a series of fouling and cleaning experiments. Their study has proven that cleaning performed with a cleaning intensity Ct between 0.5 and 1.0 g h L^–1^ improves the membrane functionality. However, the study showed that cleaning efficiency does not depend on the cleaning time, and a short intensive cleaning is better than a long comprehensive one. Regula et al. support this in their study; optimum cleaning time depends on the concentration and the study parameters (membrane material and nature of fouling) [88].

### 4.2. Temperature of the Cleaning Process

The temperature may affect membrane cleaning in three ways:

- By changing the chemical reaction balance,
- By changing the solubility of fouling materials and/or reaction products during the cleaning, and
- By changing the reaction kinetics.

Examination of the literature shows that the cleaning process at high temperatures is more effective in chemical cleaning [44,74]. Ahmad et al. [79] noticed that different temperatures had a significant effect on cleaning and flux recovery. In addition to this positive effect of temperature on chemical cleaning, it is also important to examine the effects of temperature on the chemicals and membranes.

Madaeni et al. [71] determined that temperature plays a crucial role in flux recovery, with a higher temperature providing a higher flux recovery. Gan et al. [55] also support Madaeni’s findings. They demonstrated that increased temperature also has a significant effect on the level of stable water flow recovery along with the full cleaning time, which is also important in terms of cost. They also pointed out that the increase in cleaning rate with temperature is more pronounced at a lower temperature range of Tc < 40 °C. The value of FR stat at Tc = 80 °C was approximately 20% higher than the value at Tc = 22 °C.

Kim et al. [85] studied the effect of the temperature of the chemical cleaning process on flux recovery. Their study showed that the flux at 60 °C was more than at 25 °C. They observed that the flux doubled at 60 °C. This implies that an increase in temperature has a significant effect on flux. They also pointed out that cleaning at high temperatures does not have any side effects.

Bartlett et al. [89] examined the effect of temperature on flux recovery in a range of 30–70 °C. In their study, they used optimum sodium hydroxide concentrations of 0.2% by weight for a sintered stainless-steel membrane and 0.4% by weight for a ceramic membrane. A temperature of 50 °C was discovered to be the optimum temperature for both cases. They also stated that increasing the temperature further causes the flux recovery to decrease. Furthermore, an increase in temperature causes the maximum flux recovery time to be shortened. They stated that a time of 8 min at 40 °C for the sintered stainless-steel membrane decreased to 30 s by increasing the temperature to 70 °C.

### 4.3. Concentration of Chemicals

Membrane cleaning is based on chemical reactions between cleaning chemicals and fouling materials. The concentration of cleaning chemicals may have an effect on both reaction equilibrium and reaction rate. The concentration of cleaning chemicals is important in order to maintain the optimum reaction rate. However, it also has an important effect on overcoming the mass transfer barriers of fouling layers. In practice, the chemical concentration should be high enough to provide the desired reaction rate. Mass transfer determines the limited concentration sufficient for cleaning [44].

Madaeni et al. [58] studied chemical cleaning for PVDF microfiltration membranes to remove whey proteins. However, they examined cleaning parameter such as cleaning time and cleaner concentration. The results showed that the concentration of cleaner had an effect on cleaning performance. They determined that for acid and alkaline, a higher cleaner concentration increases the efficiency of cleaning. They stated that this also applies to surfactants.

Xing et al. [90] investigated the fouling and cleaning of a tubular microfiltration membrane for municipal wastewater reclamation. In this study, they also studied the optimization of the alkaline concentration for cleaning efficiency. The residual permeability was 4% and 6% for groups of 0.2% NaClO and 0.3% NaClO, respectively. However, after 15 min of alkaline cleaning, the permeability was 50% and 35%. Hence, the results show that a relatively low concentration of alkaline cleanser was even more effective than a high one.

Garmsiri et al. [34] stated that a low concentration provided better flux recovery. In their study, they used a triple SDS + EDTA + NaOH solution with a concentration of 5 mM as the cleaning agent and found that the flow recovery was 35.895% and 49.322% in the first and second cleaning stages, respectively. When they increased the concentration of the cleaning agents to 10 mM, they found that in the first and second cleaning stages, the flow recovery was reduced by 14.318% and 19.019%, respectively.

Ahmad et al. [79] studied the chemical cleaning of cellulose acetate (CA) microfiltration membrane fouled by microalgal biomass. They also evaluated the best concentration of cleaning agents for chemical cleaning. Four different concentrations were studied (0.1%, 0.5%, 0.75%, and 1.0%). They noted that a higher concentration of NaOCI provides a higher Jp. They also found that this effect was limited; however, this observation was valid only for concentrations below 0.75%.

Kim et al. [85] examined the effects of chemical agents on flux recovery in their study. They observed that the flux value increased as the NaOH concentration increased in the range of 1–3%. Maximum flux recovery was at the level of 3–4%. The highest flux recovery was achieved with the use of 3% NaOH. This is 92% of the initial water flux. They stated that flux recovery decreased at concentrations above 5% NaOH. They stated that flux recovery decreased at concentrations above 5% NaOH.

Membrane cleaning is an important part of membrane technology since it affects membrane performance, life-span, and energy demand. The current strategies for the cleaning process are not enough to achieve full flux recovery. For a better understanding of the cleaning process, deep research is needed. Herein, we focused on the chemical cleaning process and parameters by including current chemical cleaning methods. All the studies indicate that there is a need for optimization of cleaning agents and cleaning conditions.

## 5. Conclusions

Over the last few decades, membranes have gained importance in separation technologies, particularly due to the fact that they have high permeability and uniform surface, they are easy to operate, no chemical knowledge is needed, there is no requirement for a large amount of space, and they are easy to scale up. The greatest disadvantage of the use of membranes is their fouling. Based on their structure, the foulants may diffuse through the pores and clog them or they may accumulate on the surface and create a cake layer, which may cause a reduction in the permeability of the membrane. Impressive progress has been achieved in the design of membranes that may reduce fouling. However, a continuous cleaning process is still needed to improve membrane life-span and permeability. The cleaning process and selection of cleaning agents depend on the membrane type and the type of foulants. Physical and chemical cleaning are two types of membrane cleaning processes selected based on the fouling removal mechanism. Physical cleaning is mainly used for the removal of reversible fouling such as a cake layer, whereas chemical cleaning is used for irreversible fouling using chemical agents. Even though chemical cleaning mostly helps permeability recovery, the membrane may become damaged by incorrect chemical cleaning; therefore, it is important to determine the right cleaning agent according to the foulants and membrane type.

This article reviews and discusses the fouling and cleaning process in microfiltration membranes. Fouling is a problematic phenomenon in which microfiltration membranes have been struggling for a while, and extensive research needs to be done on this issue. A better understanding of the fouling mechanism, cleaning mechanism, and cleaning agents on various foulants, membrane types and modules, and cleaning conditions is required to improve membrane performance and reduce costs. Membrane fouling can generally be managed in two ways, one of which is to minimize the contamination rate and the other is to clean the membrane. It is known that physical cleaning is unsuccessful for cleaning of irreversible contamination on its own, although the chemical cleaning methods are successful. From this point of view, we tried to review the chemical cleaning process, which is more challenging than a physical one. On the other hand, the chemical cleaning process causes pollution and not an ecofriendly method. Self-cleaning membranes can be recommended as a more sustainable and ecofriendly solution for future studies.

## Figures and Tables

**Table 1:** Process of membrane technology and properties of membranes.

Membrane Process	Properties
Separation	Liquids, particles, molecules, ions, gases, etc.
Driving forces	Pressure, concentration, temperature, voltage
Configuration	Hollow fibers, flat sheets, tubes, capillary
Structure	Charged, solid, porous
Morphology	Asymmetric, symmetric

**Table 2:** Membrane separation process according to the pore size of the membrane (Adapted from [20]).

Process	MF	UF	NF	RO
**Selectivity**	0.1–5 µm	0.01–0.1 µm	0.001 to 0.01 µm	0.0001 to 0.001 µm
**Separation mechanism**	Molecular sieve	Solution diffusion	Molecular sieve	Solution diffusion
**Material retained**	Suspended particles, bacteria	Micropollutants, salt, glucose, lactose	Macromolecules, colloids	Dissolved salts
**Material passed**	Water, dissolved solutes	Water, monovalent salts	Water, dissolved salts	Water

**Table 3:** Updated cleaning studies in microfiltration during the past 5 years.

Materials	Foulant	Cleaning Type	Results	References
Mullite ceramic microfiltration membrane (MF)	Crude Oil	Two-step chemical cleaning: Acid (sulfuric acid (H2SO4)), surfactant (sodium dodecyl sulfate (SDS)), chelating agent (ethylene diamine tetraacetic acid (EDTA)), and alkaline (sodium hydroxide (NaOH))	EDTA and SDS with a concentration of 5 and 10 mM were the best cleaning agents which have flux recovery of about 31.265% and 57.778% Binary solution of SDS + EDTA with the concentration of 5 mM was the best cleaning agent among binary and ternary cleaning solution agents, which led to 41.802% and 65.163% flux recovery	[34]
PVDF MF membranes	Organic matter in municipal wastewater	Physical Cleaning: Granules (polyethylene glycol cylindrical granules) and vibration of membrane modules Chemically enhanced backwash (CEB): citric acid (1% (*w*/*v*)) was used for CEB.	The single cleaning method did not work. A combination of membrane vibration and agitation of the tank was found to be effective. Physical cleaning efficiently mitigates reversible membrane fouling, and CEB was performed very well for control of irreversible fouling.	[35]
PVDF MF membrane	Pomegranate juice	Chemical Cleaning: Various solutions including water (with 0.5, 1, and 1.5% NaOH or 0.1% hydrochloric acid), ethanol (with 77% and 96% purity), and mixture of ethanol (77%) with acetic acid (96%) with 99:01 ratio	The ethanol 77% showed the best performance among different solutions for cleaning	[36]
Ceramic MF membrane	Cactus juice	Ultrasonic dental scaler (UDS): operated for 30 min at 29 kHz. Chemical cleaning: s (NaOH and NHO3) at high temperatures (50 and 80 °C), followed by rinsing with pure water	Water flux after cleaning with ultrasound was lower (74.4% at 0.3 bar and 67.74% at 0.5 bar) than the water flux obtained by chemical cleaning	[37]
Flat sheet MF membranes with supporting fibers (polyethylene terephthalate (PET))	Microalgae Nannochloropsis salina	High-Pressure Jet Cleaning: The angle of 70° at a pressure of 130 bar and a cleaning duration of 10 s	This method was restored about 80% of the initial throughput of the membrane. Higher pressure and a longer cleaning duration are supported by a higher throughput of the membrane	[38]
Polytetrafluoroethylene (PTFE) MF membrane	Raw wastewater (contained a mass of anionic polyacrylamide (APAM) with the concentration of 749 ± 33 mg L^−1^, which was used as oil displacement and contained organic matter in large quantity, suspended solids (SS), and salt.	Chemical cleaning: NaOH, NaClO, HCl, HNO3, SDS, and EDTA solutions with a concentration of 0.5% (wt %) were used separately for immersion at 40 °C for 3 h	The cleaning efficiency of 93 percent was achieved by mixing with 0.04 N NaClO + 200 mg L1NaOH, which was found to be better than individual cleaning. Consecutive cleaning with NaClO + NaOH–HCl has also restored 98 percent of the membrane. In addition, the cleaning temperature and time were set at 40C and 3 h.	[39]
MF ceramic membrane made from Sayong ball clay	Natural organic matter (NOM)	Chemical Cleaning: NaOH cleaning Membrane sintered at 1050 °C and at a temperature of 25 °C and TMP of 1 bar during filtration	Flux rate improved 1.8 times after chemical cleaning with 0.1M NaOH	[29]
MF Membrane	Effluent organic matters (EfOM) (feed waters containing (i) single foulant and (ii) mixed foulants of humic acids, polysaccharides, and protein)	Salt cleaning	The salt cleaning efficiency tends to be more effective for fouling caused by feed water containing more polysaccharide than the other foulants	[40]

**Table 4:** Various fouling mechanisms according to fouling types.

Fouling Type	Fouling Mechanism
Colloidal Fouling	Pore Narrowing, Pore Plugging
Organic Fouling	Pore Narrowing, Gel/Cake Formation
Inorganic Fouling	Pore narrowing, Gel/Cake Formation
Biofouling	Pore Narrowing, Pore Plugging, Gel/Cake Formation–most prominent

**Table 5:** Common foulants in microfiltration applications and related references.

Process	Foulant	Reference
Sterile filtration	Cells, Proteins	[42]
Beer clarification	Macromolecules of proteins and polysaccharides, minerals, cell debris, protein-polyphenolic aggregates (chill haze),	[27,28]
Whey	Protein, fat, ash, lactose, and moisture.	[29,30]
Wine clarification	Polysaccharides, polyphenols, and tannic acid	[43]
Skim milk filtration	Proteins, minerals, carbohydrates	[44]
Oil-in-emulsion filtration	Oil droplets	[45]
Cell microfiltration	Protein aggregates	[46]

**Table 6:** Common cleaning agents.

Type of Cleaning Agent	Typical Chemical	Properties	Reference
Caustic	NaOH	Removal of organic (e.g., polysaccharides) and microbial foulants, hydrolysis, solubilization	[66]
Alkalis	Carbonates, hydroxides, phosphates	Alteration of surface charges, pH regulation, decrease in the number of bonds between the foulant and the membrane surface	[41,54]
Acids	Sulfuric, nitric, citric, and phosphoric (HCl, HNO3, H2SO4, H3PO4, citric, oxalic)	Remove common scaling compounds and metal dioxides, dissolve inorganic precipitates; some acidic hydrolysis of macromolecules (such as: polysaccharides and proteins)	[25,45]
Enzymes	Proteases and lipases (a-CT, CP-T, peroxidase)	Hydrolyze e.g., proteins, lipids	[45,52]
Surfactants	Anionics, nonionics, cationics (alkyl sulphate, SDS, CTAB)	Dispersion, emulsifying, surface conditioning (modify the surface charge, increase wettability), disrupt functions of bacteria cell walls	[25,41]
Sequestrants	Ethylenediamine tetra acetic acid (EDTA), polycarboxylate	Removal of mineral deposits	[67]
Disinfectants (and oxidants)	Metabisulphite, NaOCl, peroxyacetic acid, hydrogen peroxide (H2O2), chlorine, and hypochlorite	Increase in hydrophilicity, oxidation of organics, destruction of pathogenic micro-organisms.	[47]

**Table 7:** Membrane types and chemical cleaners.

Material	Type of Membrane	Type of Foulant	Cleaning Chemical	Results	References
PET	Hollow fiber MF	Oil from contaminated seawater	Caustic soda, oxalic acid, and sodium hypochlorite	As compared to acid cleaning, alkaline cleaning showed a higher recovery of operating cycle time but a lower recovery of permeate flux. The best-operating cycle time and flux recoveries were achieved using a mixture of alkaline and acid cleaning agents (e.g., 96 percent and 94 percent, respectively).	[68]
Ceramic	MF	WPC (whey protein concentrate) powder	Sodium hydroxide (NaOH purity > 99%) (cleaning time (between 5 and 45 min) and transmembrane pressure (from 0.25 to 0.84 bar))	The bulk of protein fouling was removed within the first few minutes, and the recovery of the flux reached the plateau at a cleaning time of approximately 5 min.	[58]
PVDF	MF	Whey	HCL, NaOH, Triton-X100 (cleaning time (30 min.), stirring speeds (400 rpm.) without applying pressure)	Acids showed more efficiency than alkaline to remove mineral compounds.	[68]
Ceramic	CF-MF	Commercial rough beer, beer type A	NaOH, HNO3, Ultrasil 11 (low transmembrane pressure Δp = 0.2 bar, a cross-flow velocity v = 2 m/s (corresponding to Reynolds number Re = 1552), and a constant cleaning temperature Tc 22).	Sodium hydroxide was found to be of the highest cleaning power among the three types of chemicals.	[74]
Sintered stainless steel	MF	WPC	NaOH (cleaning time (30 min.), temperature (50 °C), TMP (0.5 bar) and a CFV (1.6 ms^−1^)).	An optimum concentration was found as 0.2 wt in a low percentage of flow recovery.	[65]
PVDF	MF	Organic matter (15 mg/L humic acid), with inorganic matter (1 mg/L Fe and 1 mg/L Mn) and a mixture of organic and inorganic matter (humic acid, Fe and Mn)	NaOH and citric acid solution	The cleaning efficiency was different by changing the two chemicals’ cleaning sequence (acid/base and base/acid). Flux recovery was found 20 percent higher in the base/acid sequence.	[75]
PVDF	Hollow module MF	The raw water from the first tank of the Guui pilot plant (i.e., Feed 1) shows relatively low turbidity of 12–55 NTU and a moderate DOC concentration of 2.6–3.0 mg/L. The other feed water (i.e., Feed 2) was collected from the second tank of the plant, to which the concentrate from the first tank was introduced. Feed 2 contained highly concentrated turbid matter, i.e., 343–678 NTU, and DOC compounds, i.e., 5.7–7.8 mg/L. The pH was in the range of 7.1 and 7.5 for Feed 1 and 7.7–8.0 for Feed 2.	NaOCl and NaOH, citric acid	The chemical cleaning procedures resulted in 0.93 of the recovery of the water flow for Feed 1 and 0.74 of the recovery of the water flow for Feed 2.	[76]
GVWP PVDF	MF	Raw milk	Nitric acid Hydrochloric acid Sulfuric acid Sodium hypochlorite Sodium hydroxide Potassium hydroxide Ammonium hydroxide Ammonia Ethylenediaminetetraacetic acid (EDTA) Sodium dodecyl sulfate (SDS) Triton X-100 Phosphoric acid 2-Propanol	SDS had superior results either alone or in combination with NaOH as a powerful cleaning agent and EDTA as a chelating agent. The cleaning efficiency of hydrochloride and nitric acids was poor. Sodium hypochlorite as a strong base showed a suitable result for chemical cleaning of protein. It was concluded that EDTA could not be used as a chemical cleaner by itself.	[71]
PVDF	Durapore^®^ membrane was used (flat sheet PVDF from MilliporeTM with nominal pore size of 0.22 m)	The model solution was prepared with 3.5 g/L of sodium alginate and 2 g/L of BSA for the single cleaning, and 1 g/L of alginate + 1 g/L of BSA for the other experiments.	NaOCl	Cleaning efficiency varied between single and cyclic (i.e., repeated fouling/cleaning cycles): 1% of NaOCl achieved 95% efficiency in a single cleaning, while only 87% cleaning efficiency was seen during cyclic cleaning	[77]
Polyolefin with a pore size of 0.4 µm	MF Membrane	Glass industry wastewater	Ultrasound and Chemical cleaning (EDTA, citric acid, NaOH)	Sonication in a caustic solution achieved maximal flux recovery of more than 95%.	[78]
Cellulose acetate (CA)	MF Membrane	Microalgal biomass	NaOCl, NaOH, HNO3 and citric acid	0.75% NaOCl had the best cleaning performance, and approximately 98% flux recovery was achieved. 0.75% NaOH was less effective, resulting in only 68% flux recovery.	[79]
Asymmetric multilayer Al2O3 and TiO2 ceramic	MF Membrane	Oil and grease	NaOH solution, Ultrasil P3-14, Ultrasil P3-10	The efficiency of chemical cleaning of MF and NF membranes was found in the range of 33 to 61% using various lye solutions	[80]
PS (0.1 μm) and PS (0.2 μm)	MF Membrane	Oily wastewater from the Tehran refinery	EDTA, SDS	The findings revealed that combinations of SDS and EDTA could effectively clean fouled polymeric membranes.	[81]
PP	CMF Hollow Fibre MF	Organic and biological fouling	*Memclean (proprietary ingredients 25% *w*/*w*, citric acid 10% *w*/*w*, and water balance), *Lavasol II (High pH 10.5–12, cleaner for removing organic and biological foulants, Phosphate free), *Lavasol IV (Neutral pH 4.5–6.5, cleaner for removing organics and proteins), *Lavasol MF (High pH 10.5–12, cleaner for removing organic and biological foulants), *Minncare (Peracetic acid solution oxidizing agent, oxidizes microbial cell proteins and enzyme systems for biofilm removal, decomposes into oxygen, water, and acetic acid)	Caustic soda, a high pH commercial cleaning solution called Memclean C, and hydrogen peroxide were the best cleaning solutions for extracting organic and biological foulants from membrane fibers and restoring membrane performance.	[82]
Tubular ceramic	MF	WPC	Sodium hydroxide (NaOH purity 99%, SDS); nitric acid	It has been observed that sodium hydroxide provides flux recovery through desorption and solubilization of proteins, while nitric acid has a detrimental effect on membrane resistance.	[69]
PVDF	MF, UF	Skimmed milk	Sodium hydroxide, hydrochloric acid, citric acid	Chemical cleaning, used in this study, damaged the membranes.	[70]
Flat-sheet (PVDF)	MF	Milk solution 1 w%	Chemical cleaning (EDTA) and, ultrasound cleaning	Mixed wave ultrasound had a higher cleaning efficiency than other treatments, whether used alone or in combination with EDTA 1 mMole. There was a synergistic effect when ultrasound was used with EDTA as a cleaning factor.	[45]
PVDF	MF	α-lactalbumin powder	Chemical cleaning (NaOH) and, Rinsing	The maximum flux recovery achieved by rinsing only about 6% of pure water flux. Flux recovery increased by up to 90% after the caustic solution was added, indicating that almost all of the remaining deposits inside the pores were cleaned as well.	[83]
PVDF	MF	Humic acid (HA)	NaCl	High flux recovery of 94.20% was obtained at NaCl concentration of 100 mM with an agitation speed of 600 rpm and temperature of 35 °C.	[84]
PE	MF	Oil from contaminated seawater	Caustic soda, oxalic acid, and sodium hypochlorite	Alkaline cleaning recovered more operating cycle time but less permeate flow than acid cleaning. The best working cycle time and flux recovery were achieved using a combination of alkaline and acid cleaning agents (e.g., 96% and 94%, respectively).	[68]
Mullite ceramic	MF	Oily wastewater	Acid (sulfuric acid (H2SO4)), surfactant (sodium dodecyl sulphate (SDS)), chelating agent (ethylene diamine tetraacetic acid (EDTA)) and alkaline (sodium hydroxide (NaOH)).	Sulfuric acid was found as the weakest agent to remove foulants. SDS with a concentration of 10 mM utilized 57.78% flux recovery. SDS + EDTA solution with a concentration of 5 mM was the best cleaning agent between the dual and triple cleaning agents, providing a flow recovery of 41.802% and 65.163%, respectively.	[34]
Stainless steel tubular membrane, 316 L stainless steel tube surface-coated with a sintered TiO2 layer	MF	Terephthalic acid solids	Sodium hydroxide (NaOH), Ultrasil 10 (Henkel), sodium dodecyl sulfate (SDS), and Tween 80	Flux recovery increased when the NaOH concentration raised above the range of 3-4 percent (w/v) NaOH but decreased when the NaOH concentration grown above 4 percent. The addition of surfactants (SDS and Tween 80) to the caustic cleaning agent resulted in a significant reduction in cleaning efficiency.	[85]
PAN	MF	Activated sludge and yeast suspension	NaOCl, SDS, and NaOH	When compared to SDS and NaOH, the cleaning efficiency of NaOCl was found to be superior.	[72]
Ceramic	MF	Coke particles, oily wastewater	0.1 M HCl, 0.1 NaOH, and 1 wt.% SDS	The best cleaning agent was 0.1 M NaOH solution, which provided the highest flux recovery (80%). As a result, NaOH provided a normal flux recovery, while HCl failed to provide an adequate flux recovery.	[86]

## Data Availability

Not applicable.
